# *In silico* prediction of molecular mechanisms of toxicity mediated by the leptospiral PF07598 gene family-encoded virulence-modifying proteins

**DOI:** 10.3389/fmolb.2022.1092197

**Published:** 2023-01-23

**Authors:** Reetika Chaurasia, Joseph M. Vinetz

**Affiliations:** Department of Internal Medicine, Section of Infectious Diseases, Yale University School of Medicine, New Haven, CT, United States

**Keywords:** cytotoxin, lectin, ricin B chain, CARDS toxin, virulence factor, *Leptospira interrogans*, microbiology

## Abstract

Mechanisms of leptospirosis pathogenesis remain unclear despite the identification of a number of potential leptospiral virulence factors. We recently demonstrated potential mechanisms by which the virulence-modifying (VM) proteins—defined as containing a Domain of Unknown function (DUF1561), encoded by the PF07598 gene family—found only in group 1 pathogenic *Leptospira*—might mediate the clinical pathogenesis of leptospirosis. VM proteins belongs to classical AB toxin paradigm though have a unique AB domain architecture, unlike other AB toxins such as diphtheria toxin, pertussis toxin, shiga toxin, or ricin toxin which are typically encoded by two or more genes and self-assembled into a multi-domain holotoxin. Leptospiral VM proteins are secreted R-type lectin domain-containing exotoxins with discrete N-terminal ricin B-like domains involved in host cell surface binding, and a C-terminal DNase/toxin domain. Here we use the artificial intelligence-based AlphaFold algorithm and other computational tools to predict and elaborate on details of the VM protein structure-function relationship. Comparative AlphaFold and CD-spectroscopy defined the consistent secondary structure (Helix and ß-sheet) content, and the stability of the functional domains were further supported by molecular dynamics simulation. VM proteins comprises distinctive lectic family (QxW)_3_ motifs, the *Mycoplasma* CARDS toxin (D3 domain, aromatic patches), C-terminal similarity with mammalian DNase I. *In-silico* study proposed that Gln412, Gln523, His533, Thr59 are the high binding energy or ligand binding residues plausibly anticipates in the functional activities. Divalent cation (Mg^+2^-Gln412) and phosphate ion (PO_4_]^−3^-Arg615) interaction further supports the functional activities driven by C-terminal domain. Computation-driven structure-function studies of VM proteins will guide experimentation towards mechanistic understandings of leptospirosis pathogenesis, which underlie development of new therapeutic and preventive measures for this devastating disease.

## Introduction

Leptospirosis is a globally neglected zoonotic disease caused by pathogenic spirochetes of the genus, *Leptospira*, which has a conservatively estimated global burden of disease of more than 1 million cases and 58,900 deaths (Costa et al., 2015; Torgerson et al., 2015), comparable to other major tropical diseases such as dengue, cholera, typhoid fever, and visceral leishmaniasis (Hartskeerl et al., 2011; Picardeau, 2015; Torgerson et al., 2015; Goarant, 2016; Rudd et al., 2020). People admitted to hospital with severe leptospirosis develop life-threatening disease with multi-organ damage including jaundice, acute kidney injury and shock (Weil’s disease). The most feared complication is pulmonary hemorrhage (Sehgal et al., 1995; Ko et al., 1999; Marotto et al., 1999; Segura et al., 2005; Andrade et al., 2007; Gouveia et al., 2008; Wagenaar et al., 2010; Truong and Coburn, 2011; Helmerhorst et al., 2012; Ruwanpura et al., 2012; Ellis, 2015). Histopathological analysis of lung tissues in severe pulmonary leptospirosis syndrome finds little leptospiral antigen or intact *Leptospira* (Nicodemo et al., 1997; Nicodemo and Duarte-Neto, 2021), but rather reveals damage to alveolar epithelial and activation of endothelial cells; which explain the deposition of immunoglobulin and complement as probably secondary events (Nicodemo et al., 1990; Nicodemo et al., 1997; Nally et al., 2004; Croda et al., 2009; Croda et al., 2010; De Brito et al., 2013a; Nicodemo and Duarte-Neto, 2021); some have suggested that a leptospiral “toxin” may mediate pathogenesis (Adler and de la Pena Moctezuma, 2010; Aagaard et al., 2012; Goncalves-de-Albuquerque et al., 2012; Chaurasia et al., 2022a). Pathogenic *Leptospira* express a wide range of putative virulence factors such as secretory proteins, adhesins, enzymes, and surface proteins which allow them to establish an infection in host cells and avoid an immune response (Ko et al., 2009; Murray, 2015; Grassmann et al., 2017; Picardeau, 2017; Daroz et al., 2021; Samrot et al., 2021). Recent insights into the pathogenesis of leptospirosis and characterization of these virulence factors identified ricin B lectin-like AB cytotoxins, sphingomyelinases, collagenase, pore-forming toxins and other potential virulence factors (Lee et al., 2002; Vieira et al., 2013; Picardeau, 2017; Chaurasia et al., 2018; Chaurasia and Sritharan, 2020; Chaurasia et al., 2022a). These and likely other virulence factors are essential for the dissemination of pathogenic *Leptospira* and are potentially involved in cell lysis, tissue damage, and manifestations of severe disease. Secreted cytotoxins are typical virulence factors of numerous bacterial pathogens which disrupt epithelial barrier function, damage cells, and activate or modulate host cellular and immune responses (Lubkin and Torres, 2017; Carlini et al., 2021). Efforts to understand the molecular and cellular pathogenesis of leptospirosis remain in their infancy and approaches to prevent leptospirosis or ameliorate its pathogenesis are based on mechanistic understandings of the biology of *Leptospira*-host interactions.

A diverse PF07598 paralogous gene family encoding Virulence-Modifying (VM) proteins were first identified in a pathogenomic screen of *L. interrogans* serovar Lai (Lehmann et al., 2013). This gene family was found to have orthologs present only in Group 1 pathogenic *Leptospira* and expanded in *L. interrogans, L. kirschneri, and L. noguchii*, including the cosmopolitan and lethal *L. interrogans* serovars Copenhageni and Canicola (Lehmann et al., 2013; Lehmann et al., 2014; Fouts et al., 2016). The absence of the PF07598 gene family in intermediate or saprophytic *Leptospira* supports a role of VM proteins as virulence factors (Lehmann et al., 2013; Fouts et al., 2016). VM proteins are secreted exotoxins with encoded secretory signal peptides, two N-terminal tandemly repeated R-type lectin domains (RBLs) (except for one ortholog of LA0591 that lacks the RBLs), and a C-terminal toxin domain with DNase activity (Fouts et al., 2016). PF07598 genes are upregulated both *in vitro* under conditions mimicking the *in vivo* host environment (Matsunaga et al., 2007) and *in vivo* in small animal models of acute infection (Lehmann et al., 2013); supporting the hypothesis that they are involved in leptospirosis pathogenesis.

VM proteins, with the exception of natural CBR deletion variants (LA0591, ∼313 amino acids, and its orthologs), belong to the classical AB toxin paradigm (Chaurasia et al., 2022a). PF07598 genes are transcribed from a single gene and translated into an all-in-one multi-domain protein; this structure is distinct from most other bacterial AB toxins such as diphtheria toxin (*Corynebacterium diphtheriae*: AB (Murphy, 2011)), pertussis toxin (*Bordetella pertussis*: A (S1)-B (S2-S5) (Stein et al., 1994)), shiga toxin (*Shigella dysenteriae*: AB5 (Johannes, 2017)), exotoxin A (*Pseudomonas aeruginosa* AB (Ogata et al., 1990)), and castor bean-derived ricin toxin *Ricinus communis*: AB (Lord and Spooner, 2011), which are typically encoded by two or more separate genes. AB toxins contain at least one subunit or polypeptide (B chain) that recognizes a specific receptor on the cell surface and one subunit or polypeptide (A chain) that enters the cell to exert its effect on one or more target proteins (Cherubin et al., 2018). From the genomic perspective, across the entire clade of group 1 pathogenic *Leptospira,* VM proteins are predicted to contain two conserved (∼78% average pairwise amino acid identity), tandemly-arrayed β-trefoil-fold N-terminal ricin B-like lectin domains, (binding and internalization), and a less conserved (∼63% average pairwise amino acid identity) C-terminal toxin domain with DNase activity (intracellular trafficking/enzymatic activity) (Chaurasia et al., 2022a).

Here we use AlphaFold, an artificial intelligence-based computational approach, combined with other *in silico* tools and validated by *in vitro* studies, to provide further insight into the structure-function relationships of VM protein domains, focusing on conserved, functional sequence motifs, the significance of disulfide bonds, hot-spots of potential ligand-receptor interactions and active site residues. Structure-based understandings of mechanisms by which the VM proteins encoded by the PF07598 gene family exert their effects will lead to insights into their role in both leptospirosis clinical pathogenesis and leptospiral fundamental biology.

## Materials and methods

### Structure prediction and validation of VM proteins

AlphaFold algorithm-derived structural models of leptospiral VM proteins have been reported previously (Chaurasia et al., 2022a; Chaurasia et al., 2022b) and supported by Ramachandran plots, followed by the algorithm in Verify3D https://www.doe-mbi.ucla.edu/verify3d/(Pontius et al., 1996). The structure was further supported by the PROVE algorithm (PROtein Volume Evaluation) https://saves.mbi.ucla.edu/Jobs/1016444 /prove/PROVE_PLOT.ps and Z-score mean, Z-score std-dev and Z-score RMS were calculated (Supplementary Figure S1). The figures incorporated into the current study were generated using Adobe illustrator version 25.2.

### Molecular dynamics simulation

The stability of functional domain of AlphaFold-derived VM protein (CTD of LA3490, 368aa - 639aa) were examined by molecular dynamics (MD) simulation which was performed at 100 ns using Schrodinger Desmond Maestro software (Schrödinger, New York, NY, 2021). The simulation box size was set as Standard cubical (by default) and selected based on the protein size, basically to cover all the atoms. Equilibration and MD simulation time were established to 100 ns with the 5 fs timestep. The system builder of Desmond in the Maestro program was immersed in a water-filled cubic box of 1Å spacing containing water molecules using extended simple point charge (SPC), a three-point water model with periodic boundary conditions. Nose-Hoover thermostat and Nose-Hoover barostat deterministic algorithms were used for constant-temperature and to control the pressure respectively. The total charge of the solvent system was neutralized by adding sodium (Na^+^) ions. The systems were heated linearly at constant volume (NVT ensemble) from 0 to 300 K. The equilibration was obtained at constant pressure and temperature (NPT ensemble, 300 K). All the bond-lengths of hydrogen atoms are constrained using M-SHAKE Cut-off for Van Der Waals and short-range electrostatic interactions are kept at 10 Å molecular mechanics. The charge–solvent interactions and solvent screening of charge–charge interactions significantly affect the electrostatic energies of proteins. If the atoms come closer together (so that their electron clouds overlap) the van der Waals force becomes repulsive. After the system was evaluated, the consistent compliance trajectories were taken up and analyzed to assess interoperability stability. The corresponding change of the C- spinal cord crystal structure determine the mode of selection within an MD trajectory cluster. The VSGB solvent model was used for the solvation model and the force field OPLS-2005 was used to parameterize the protein models (Li et al., 2011).

### Constructs design, protein purification and CD-spectroscopy analysis

The PF07598 gene family encoding the full-length NCBI locus tag LA3490, LA0620 and LA1402 from serovar Lai, and locus tag LIC12340 (Lai ortholog: LA1400, 97% amino acid similarity), and LIC12985 (Lai ortholog: LA0591, 99% amino acid similarity) from serovar Copenhageni and truncated 3,490 or 0,620, an N-terminal domain were synthesized with post-signal *E. coli* codon-optimized sequences. They were linked to mCherry (AST15061.1) *via* a glycine-serine hinge (GGGGSGGGGSGGGGS) except LA1400 or LA0591, were synthesized and cloned into pET32b (+) (Gene universal Inc., United States ). The soluble recombinant proteins were purified using pre-packed Ni-Sepharose AKTA Hi-TRAP column (GE Healthcare, United States ) as previously mentioned (Chaurasia et al., 2022a; Chaurasia et al., 2022b).

Full-length LA1400 and natural variant LA0591 encoding C-terminal domain without mCherry tag were dialyzed against 0.1 M borate buffer pH 8.5. Circular dichroism (CD) spectroscopy was performed with 0.2 mg/ml protein in 0.01 cm path lengths at 20 °C using a Chirascan (Applied Photophysics, United Kingdom). The spectra presented as an average of triplicate scans and were recorded from 180 nm to 260 nm at a speed of 1 nm/s. The background was corrected against the buffer blank. The data were analyzed with CDNN software in-plugged with Pro-data viewer-Chirascan analyzed the CD-spectra and determined the percentage of secondary structure content compared with AlphaFold derived structure.

### Identification and analysis of VM proteins RBL domains

To examine the structure and function of RBLs of the VM proteins, their amino acid sequences were analyzed by Predict-Protein online server (https://predictprotein.org) (Bernhofer et al., 2021). The software uses a machine learning algorithm with evolutionary information. Predict-Protein aligned 32 proteins of which 31 matches were to the PF07598 protein family. A second hit was to CARDS toxin (PDB: 4TLV, A chain), which showed considerable matches with identity 0.55, expected value: 2e-94 and match length (310 aa). The sequences of RBLs (RBL1: 41 aa-195 aa, RBL2: 196 aa-335 aa), ricin B chain (PDB: 2AAI, B chain), and CARDS toxin (PDB: 4TLV) were aligned, structurally superimposed, and visualized by PyMOL 2.4.0 (https://pymol.org/2/).

### Comparative analysis of disulfide bonds between VM proteins and ricin

The AlphaFold-derived structures of VM protein were superimposed onto ricin (PDB ID: 2AAI, A and B chain). Disulfide bond prediction, length and distance were determined using PyMOL 2.4.0. (https://pymol.org/2/).

### Demonstration of hot-spot residues and ligand binding sites in VM proteins

The analysis of hot-spot residues or ligand binding sites is often used for functional identification of amino acid residues and 3D structure-based drug discovery. The hot-spot residues or cluster of residues present at hot-spot sites makes a major contribution to the binding free energy and these sites have a high propensity for ligand binding (Li et al., 2004). The AlphaFold-derived PDB files were analyzed using online machine learning-based servers FTMap server (http://ftmap.bu.edu) (Kozakov et al., 2015), PrankWeb (https://prankweb.cz) (Jendele et al., 2019), and DeepSite (https://playmolecule.com/DeepSite/) (Jimenez et al., 2017). The FTMap server uses 16 small molecules as probes (ethanol, isopropanol, isobutanol, acetone, acetaldehyde, dimethyl ether, cyclohexane, ethane, acetonitrile, urea, methylamine, phenol, benzaldehyde, benzene, acetamide, and *N*, *N*-dimethylformamide) to identify hot-spot regions (Ngan et al., 2012; Kozakov et al., 2015).

### DNase activity and computational docking (Mg^2+^ and PO_4_^3-^) to validate DNase activity

DNase assays were performed as per our published protocol (Chaurasia et al., 2022a). Briefly, the reaction was set up with purified HeLa cell genomic DNA (QiAmp DNeasy Blood and Tissue kit; Qiagen, Invitrogen, United States), in a final volume of 15 *μ*L 1X (TM) sample buffer comprising 10 mM Tris and 3 mM MgCl_2_ (pH 7.5). Recombinant VM proteins at a concentration of 30 nM were equilibrated in TM buffer to 22 °C prior to use and later 150 ng of genomic HeLa DNA was added, and the reaction was terminated at 30 min upon addition of premixed gel loading dye (New England Biolabs, United States). Gels were stained with ethidium bromide (0.5 *μ*l/ml) and samples were analyzed by 1% agarose gel electrophoresis. The gel images were taken using Gel Doc UV illumination (Gel Logic 212 Pro, Carestream Molecular imaging, United States). The docking study was performed for the C-terminal region of LA3490 with a phosphate or magnesium ion using MGLTools 1.5.7. The size of grid box was set to 17.34 Å × 5.782 Å x 8.06 Å at x-, y-, and *z*-axis coordinates respectively. Lamarckian genetic algorithm was used as scoring function. Genetic algorithm and local search algorithm were responsible for the search of possible confirmation degree of freedom of ligands and protein.

## Results

### *In silico*, molecular dynamic simulation, and CD-spectroscopy validate the AlphaFold derived structure and recombinant protein stability

**FIGURE 1:**
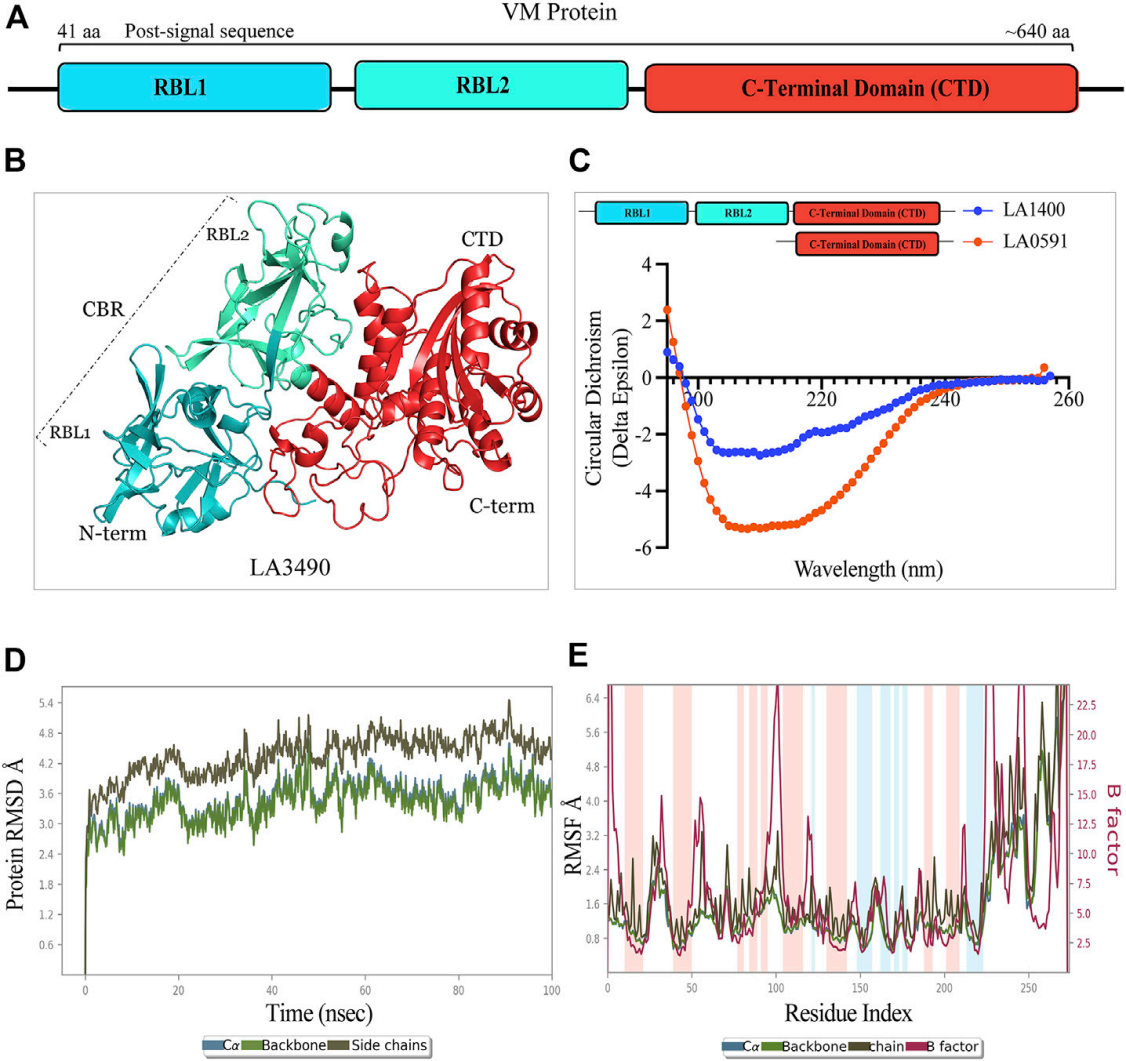
Schematic diagram, structure, and validation of VM protein by CD-spectroscopy and molecular dynamics simulation. **(A)** VM protein comprised N-terminal ricin binding domain RBL1 (41 aa-195 aa, teal color) and RBL2 (196 aa-335 aa, cyan-green color) as a carbohydrate binding domain (CBD) and the functional C-terminal domain (CTD: 368 aa-639aa, vermilion color). The domains showing amino acid numbers is with respect to LA3490. **(B)** AlphaFold derived 3D-stucture showing multi-globular domain of LA3490 VM protein **(C)** CD-spectra of soluble recombinant full-length LA1400 and natural variant LA0591 (313 aa) lacking RBLs (RBL1 and RBL2) showing the secondary (helix and ß-sheet) structure content. **(D)** The molecular dynamics simulation for CTD of LA3490 shows stabilized RMSD of an average of 3.2 Å **(E)** The RMSF plot shown 0, 1 Å and fluctuation was observed from 25 ns to 65 ns.

AlphaFold provided a detailed three-dimensional (3D) structure for five VM proteins and the structure suggest that VM proteins encode for multi-globular domains (Figures 1A,B). Ramachandran plots defined the phi-psi torsion angles for each residue and allowed delineation of the amino acid positions of AlphaFold-predicted structures (Supplementary Figure S1). These analyses were further supported by Verify3D [LA3490: 89%, LA0620: 92%, LA1402: 90%, LA1400: 98%, and LA0591; 84% of the residues have an average 3D-1D score≥0.2] (Supplementary Figure S1) (Pontius et al., 1996; Callaway, 2020; Jumper et al., 2020; Senior et al., 2020) (Chaurasia et al., 2022a). The accuracy and robustness of AlphaFold-derived VM proteins structures were experimentally validated by examining the content of the secondary structures of full-length LA1400 and LA0591, natural C-terminal variant, using CD-spectroscopy analysis. The result suggests that AlphaFold and CD-spectroscopy were comparable and shown good agreement (Figure 1C; Table 1).

**TABLE 1:** Comparative validation of secondary structure content by CD-spectroscopy and AlphaFold derived structure.

VM proteins	CD-analysis	AlphaFold
LA1400		
Helix (%)	20.9	22.51
β -sheet (%)	26.06	29.32
LA0591		
Helix (%)	35.6	34.5
β -sheet (%)	15.9	14.06

Molecular dynamic simulations were used to investigate dynamic perturbations which was carried out for protein stability conformation (Karplus and Kuriyan, 2005). It was observed that the potential energy tends to decrease for CTD of LA3490 over a 100ns simulation period, indicates that the system was stable. RMSD was calculated for protein during a simulation trajectory of 100ns to determine the average change in displacement for a specific frame about a reference frame. Root Mean Square Deviations (RMSD) graph were plotted to understand the stability the protein with backbone which are depicted in Figure 1D; these plots indicate the initial predicted conformational fluctuations observed in the system at 100ns which further stabilize in the production phase. The RMSD obtained for CTD of LA3490 is 2.4 Å, showing a gradual increase in value with fluctuation, which stabilizes at an average of 3.2 Å. Furthermore, Root Means Square Fluctuation (RMSF) of C-? atoms of all residues was also compared Figure 1E. The simulations were carried out at 100ns and the fluctuation was observed between 25ns and 65ns with 0–1 Å. The binding free energy for CTD of LA3490 was found to be −26.22 kJ mol^−1^, and energy minimization (Van der Waal −37.62 kJ mol^−1^, electrostatic −14.24 kJ mol^−^) was calculated. The polar solvation (30.6 kJ mol^−1^) and SASA (solvent accessible surface area, −9.13 kJ mol^−1^) shows the solvation energy which is required for energetic analysis of biomolecules. The overall MD simulation results showed that VM protein were stable during the entire run time.

### Characteristic carbohydrate-binding aromatic patches and the presence of (QxW)_3_ sequence motif support that VM proteins are *bona fide* R-type lectins

**FIGURE 2:**
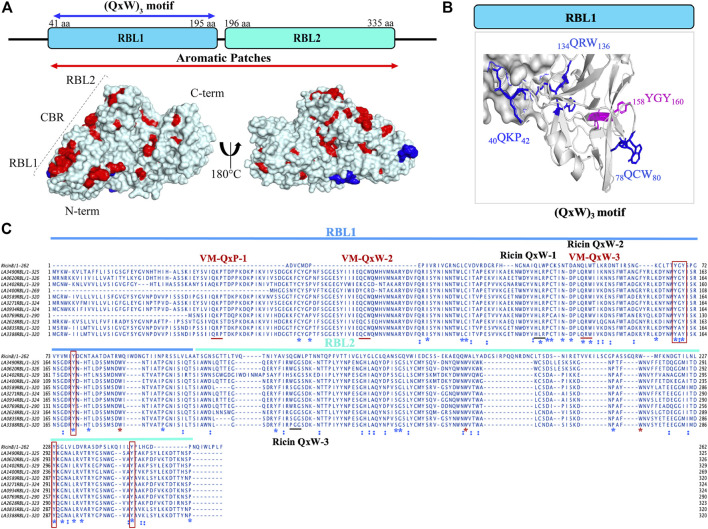
Structural and sequence representation of the (QxW)_3_ motif in VM proteins in *L. interrogans* serovar Lai. **(A)** AlphaFold-generated high-resolution 3D structure of LA3490 VM protein shows amino acids which manifest as surface aromatic patches (red color: tyrosine, phenylalanine, and tryptophan). Blue color represents the (QxW)_3_ motif within the N-terminal RBL1 domain. **(B)** RBL1 domain showing three conserved QxW motifs (blue color: _40_QKP_42_, _134_QRW_136_, and _78_QCW_80_), of which the _134_QRW_136_ motif is conserved in ricin B chain. Aromatic motif _158_YGY_160_ is highly conserved in VM proteins and in the ricin B chain. **(C)** Multiple sequence alignment was performed using MAFFT algorithm and visualized in JalView showing the conserved (QxW)_3_ motif in ricin B chain and VM proteins (red underline) from serovar Lai. RBL1 and RBL2 domains shown in blue and cyan lines respectively. Conserved sequences (identity) shown in blue *. Red boxes represent conserved tyrosine. Red * shows conserved tryptophan.

Computational analysis and *in vitro* experimentation confirmed that only the RBL1 domain (N-terminal region of LA3490, 41 aa—150 aa) structurally superimposed with the ricin B chain (PBD; 2AAI-B: seven aa to 129 aa) with a highly significant RMSD value of 1.796. RBLs as R-type lectins are predicted to be primarily involved in binding to N-terminal galactosyl-containing glycoproteins on host cell surfaces (Chaurasia et al., 2022a). RBL1 and RBL2 are rich in aromatic patches due to the presence of surface-exposed tyrosine residues, and heterocyclic phenylalanine and tryptophan amino acids (Figure 2A). These aromatic patches appear to play an important role in host receptor/carbohydrate recognition. In addition to aromatic patches, the RBL1 domain contains three conserved QxW motifs (_40_QKP_42_, _78_QCW_80_, and _134_QRW_136_) in the *ß*-trefoil fold similar to ricin B chain (Figures 2A,B). Multiple-sequence alignment of RBL1 with a ricin B chain shows that only the _134_QRW_136_ motif is conserved in both RBL1 and ricin B chain; and notably, a tryptophan is replaced by a proline in the first QxW motif (_40_QKP_42_) in the RBL1 domain (Figure 2C). The QxW sequence conserved motif plays an important role in receptor recognition and contributes to structural stabilization by hydrogen bonding within the carbohydrate-binding motif (Hazes and Read, 1995; Hazes, 1996; Hatakeyama et al., 2007). In the RBL1 domain, the sequence motif _158_YGY_160_ is highly conserved in the ricin B chain and is assumed to possess a functional binding ability similar to that of the ricin B chain (Figure 2C). LA1402 and LA1400 are ancestral VM proteins in Group I pathogenic *Leptospira* and both belong to cluster A. Computational analysis prediction suggest that these two proteins (LA1402 and LA1400) lack the _78_QCW_80_ motif; which might explain the evolution of VM protein by successive gene duplications and acquisition of the essential _78_QCW_80_ motif for binding to the host cell surface and host (Fouts et al., 2016; Chaurasia et al., 2022a).

### Sequence and structural resemblance of RBL2 domain of VM protein to CARDS toxin which rationalize the functional similarity in binding and internalization

**FIGURE 3:**
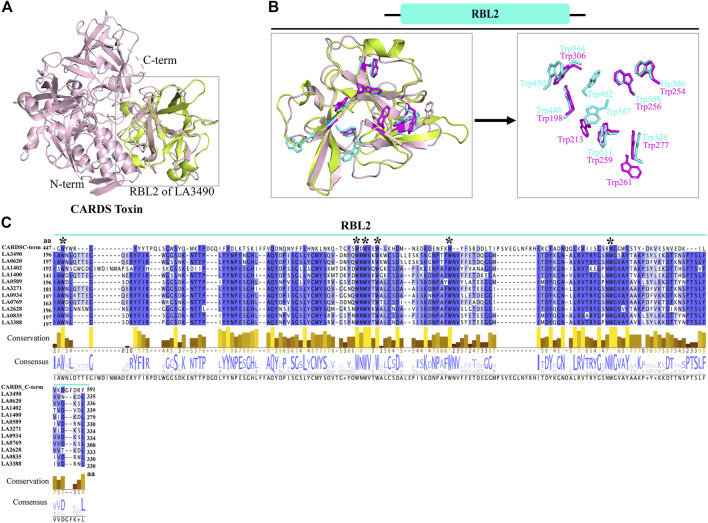
Structural and functional similarity of RBL2 of VM proteins with CARDS toxin (D3 domain). **(A)** The RBL2 domain of LA3490 VM protein (green color: 196 aa—335 aa) structurally superimposed with the C-terminal region of CARDS toxin (PDB: 4TLV, A chain, pink color) having RMSD 1.218 Å **(B)** C-terminal region of CARDS toxin (D3 domain) encodes for eight tryptophan and LA3490 VM protein encodes nine tryptophan residues. Six of the tryptophan residues are structurally superimposed in both the C-terminal region of CARDS toxin and the RBL2 domain. **(C)** Multiple sequence alignment of paralogs of RBL2 from *L. interrogans* serovar Lai was performed using MAFFT algorithm and visualized in JalView. Six conserved tryptophan residues shown by * symbol.

Superimposition of full-length VM proteins with CARDS toxin indicates that only the RBL2 (196 aa—335 aa) of VM protein was superimposed at the C-terminus of CARDS toxin (PDB: 4TLV, D3 domain: 447 aa—591 aa) with RMSD—1.218 Å (Figures 3A,B). The MAFFT (Multiple Alignment using Fast Fourier Transform) with L-INS-i (Accuracy-oriented) algorithm-based alignment between RBL2 of the VM proteins and CARDS toxin visualized in Jalview v2.11.5 (https://www.jalview.org) and suggests that the D3 domain of CARDS toxin is comprised of eight tryptophan and RBL2 contains nine tryptophan residues, and among that 6 tryptophan were conserved at the sequence and structure level in both RBL2 and CARDS toxin (Figures 3B,C). The CARDS toxins (D2+D3 trefoil) do not have galactose-binding sites, suggesting that the RBL1 of VM proteins is plausibly the sole carbohydrate binding partner. RBL2 plausibly plays a role in binding and internalization similar to the CRADS toxin.

### The architecture of intramolecular disulfide bonds similar to those of ricin toxin confirms that VM proteins are AB toxins

**FIGURE 4:**
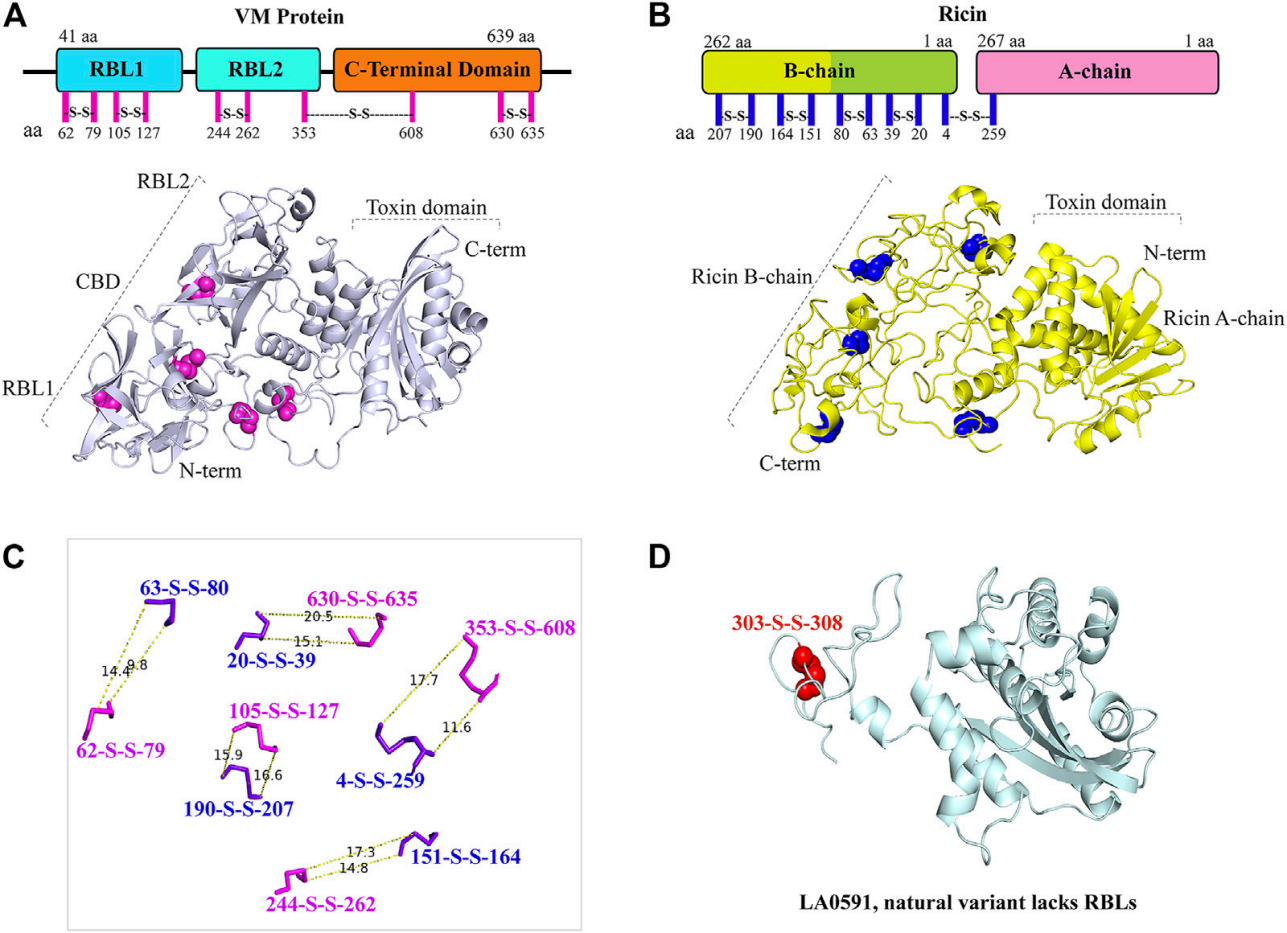
Representation and similarly of disulfide bond in LA3490 VM protein and in ricin toxin. **(A)** Schematic diagram shows the amino acid position of five disulfide bonds (magenta lines) in the VM protein domains. AlphaFold algorithm generated 3D cartoon structure of LA3490 (light blue color) showing five disulfide bonds (magenta spheres) by pairing 10 cysteine residues. **(B)** Ricin toxin (PDB: 2AAI) showing five disulfide bonds by pairing 10 cysteine residues in both schematic diagram (blue lines) and 3D-structure (blue spheres) respectively. **(C)** Measurement wizards were used to measure the distance of disulfide bond between VM protein and ricin using PyMOL 2.4.0. (https://pymol.org/2/). The dotted lines displayed the distance in Angstroms. **(D)** Presence of a single disulfide bond (red spheres) in LA0591 shown at positions Cys303-Cys308.

The AlphaFold algorithm-derived structural architecture of VM proteins shows that they contain twelve cysteine residues, of which ten cysteine are involved in the formation of five disulfide bridges similar to ricin toxin (Figures 4A–C) (Lappi et al., 1978; Chaurasia et al., 2022a). The RBL1 of VM protein contains two disulfide bonds (62 aa—79 aa, 105 aa—127 aa), the RBL2 domain are composed of one disulfide bond (244 aa—262 aa), and the C-terminal globular domain encodes a two-disulfide bond (353 aa—608 aa and 630 aa—635 aa) (Figures 4A,C). VM genes encode a single polypeptide chain, unlike ricin toxin; therefore, proteolytic cleavage of the disulfide bond plays a key role in the processing of RBL1, RBL2, and the C-terminal region into their functional domains. LA0591 lacks RBLs (RBL1 and RBL2), suggesting that this natural variant does not require binding and internalization into the host cell, so that it may be possible that this variant may be produced intracellularly which would imply a unique intracellular role in pathogenesis or possible binding to an unidentified AB partner protein. The two cysteines in LA0591 form a disulfide bond at the very C-terminus (303 aa—308 aa); and a mutagenesis study would be informative about the functional role of this disulfide bond in VM proteins and their orthologs (Figure 4D).

### Comparative computational analysis deciphering the hot-spot residues and active sites within the C-terminal domain of the VM proteins

**FIGURE 5:**
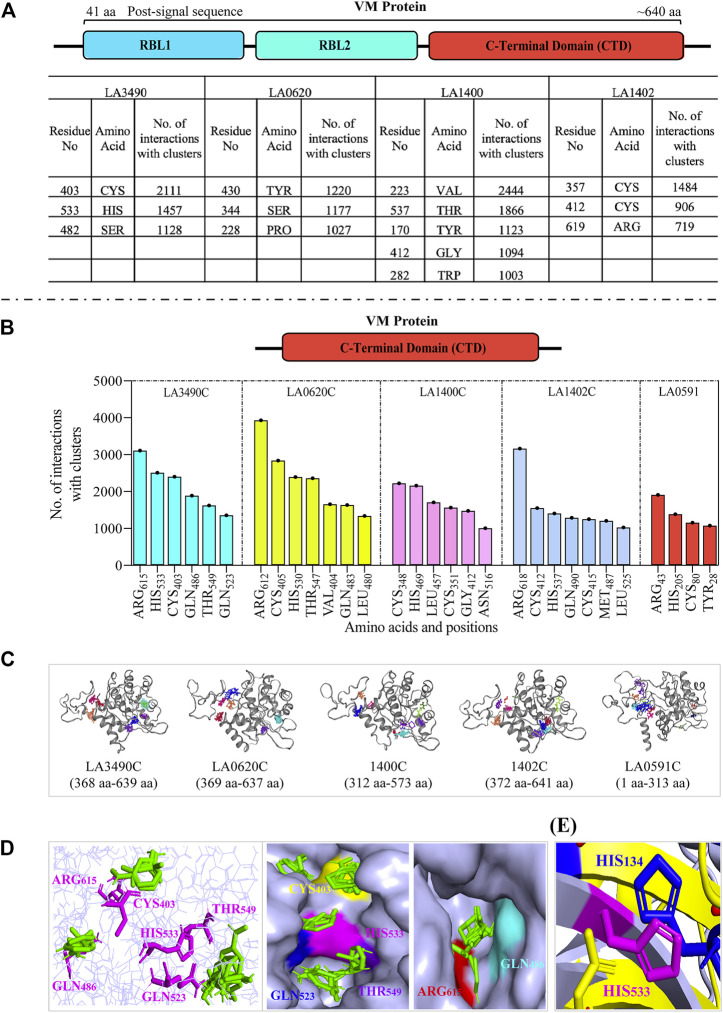
Comparative assessment of FTMap-based hot-spot and ligand binding residues in full-length and C-terminal domain (CTD) of VM proteins. **(A)** The FTMap machine learning-based algorithm using AlphaFold generated full-length VM proteins (LA3490, LA0620, LA1400 and LA1402: shown by schematic diagram) structure shows less hot-spots residues because of low binding energy and having a smaller number of interactions with clusters. Below the horizontal dash line **(B−D)** showing the analysis of carboxy-terminal domain (CTD). The schematic diagram shown the CTD domain. **(B)** Histogram showing the CTD of VM proteins (LA3490, LA0620, LA1400, LA1402, and LA0591) having higher number of hot-spots residues with high binding energy and showing a high number of interactions with clusters. **(C)** Three-dimensional view of CTD of VM proteins showing ligand binding sites. **(D)** The magenta colors are the hot-spot residues (Arg615, His533, Cys403, Gln486, Thr549, and Gln523, with respect to CTD of LA3490) shows binding with ligands (green color) in the three-dimensional view in left panel. Surface view of CTD of LA3490 shows binding of ligands with hot-spot residues in deep pockets in right panels. **(E)** Structural superimposition of CTD of LA3490 and bovine DNase (3DNI) shows the overlap of His533 (LA3490) with the catalytic residue His134 of bovine DNase.

Computational-based assessment of the AlphaFold-derived 3D structure of VM proteins (full-lengths and C-terminal domain) postulated the functionally important regions which actively participate in the substrate or ligand binding. FTMap analysis revealed that amino acids Cys403, His533, and Ser482 are hot-spot residues in the full-length LA3490 protein and showed 2,111, 1,457 and 1,128 interactions with clusters, respectively (Figure 5A). However, the C-terminal domain of LA3490 (368 aa - 639 aa) showed a higher number of hot-spot residues-interactions (Arg615-3,109, His533-2,510, Cys403-2,400, Gln486-1890, Thr549-1,622, and Gln523-1,357) with clusters (Figures 5B–D). With respect to LA3490, His533 showed high binding energy as well as consistency in other VM proteins (LA0620: His530, LA1400: His469, LA1402: His537, LA0591:His205) as a best choice hot-spot residue (Figure 5B). The current study suggests that His533 (LA3490) is a crucial amino acid, and its functional role in catalysis could be revealed by mutagenesis approaches.

**FIGURE 6:**
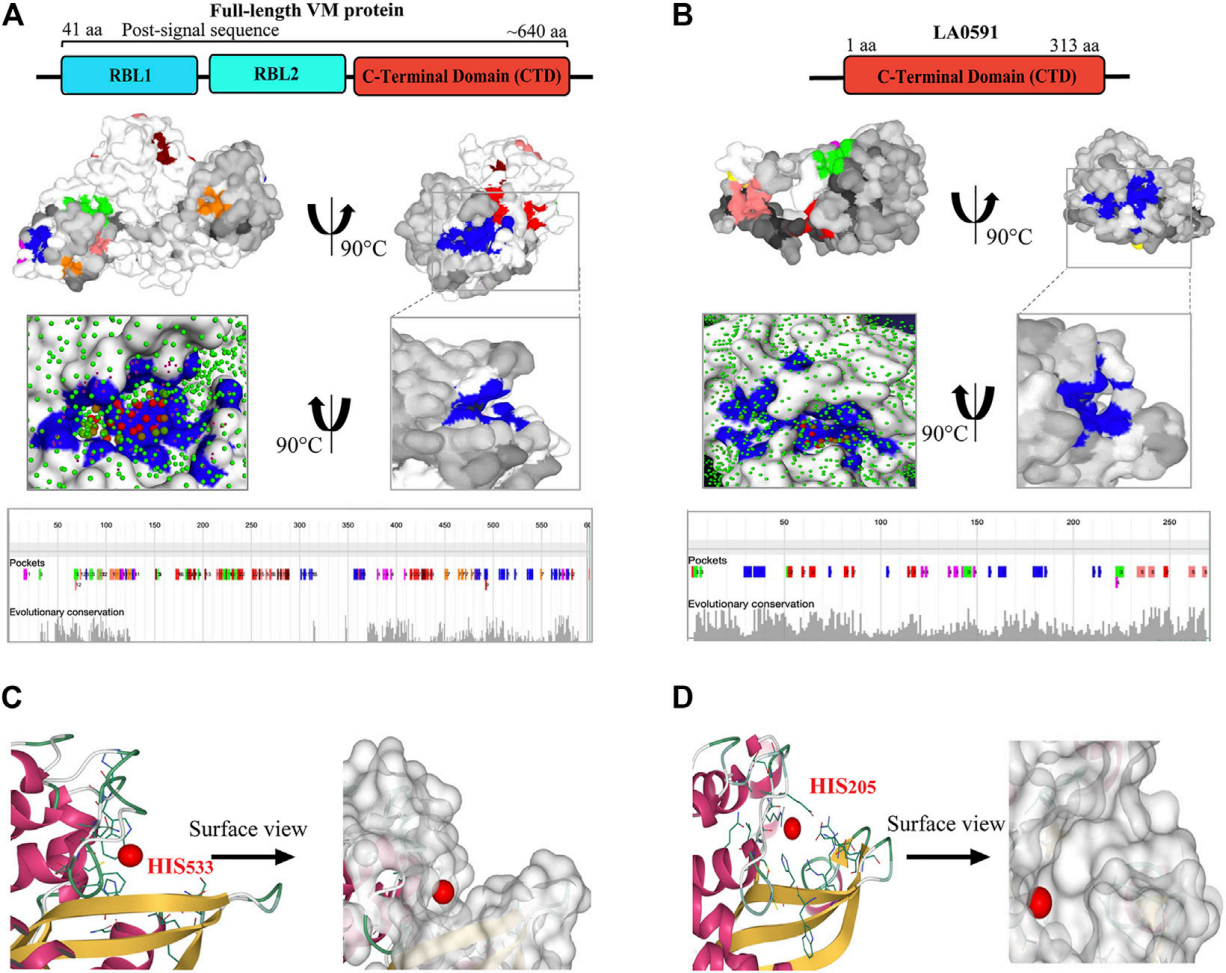
PrankWeb and DeepSite based assessment of ligand binding sites of LA3490 and LA0591 VM proteins. **(A)** AlphaFold algorithm based full-length LA3490 (shown by schematic domains) PDB file was submitted to PrankWeb. The machine learning-based tool identified 14 deep ligand binding pockets and the highest score (18.39) pocket 1 shown in blue color at C-terminal domain (CTD) with the solvent accessible surface (SAS) and the position for evolutionarily conserved pockets shown in the bottom panel. **(B)** LA0591 (schematic diagram represents the domain) shows five pockets with the highest score of 15.64, pocket 1 are shown in blue color (C-terminal) with the solvent accessible surface (SAS) and the position for evolutionarily conserved pockets is shown in the bottom panel. DeepSite machine learning-based algorithm showing the His533 and His203 residue in a deep pocket as an interactive amino acid represented on the surface view of LA3490 **(C)** and LA0591 **(D)** respectively.

PrankWeb and DeepSite are additional template-free online machine learning-based algorithms for structure-based ligand binding site prediction (Jimenez et al., 2017; Krivak and Hoksza, 2018; Jendele et al., 2019). PrankWeb identified 14 pockets in full-length LA3490, which were ranked 1 to 14 based on the probability and solvent-accessible surface (SAS points). Pocket 1 scored 18.30 with 0.817 highest probability and 106 SAS points among the remaining pockets (Figures 6A,C, Supplementary Table S1). The highest score pocket 1 is located at a C-terminal groove and encompasses amino acids Cys403, Gln523, His533, and Thr549, which were also screened by the FTMap server (Table 2). The DeepSite machine learning-based algorithm identified two deep pockets, His533, Thr549, and Gln523 located at the C-terminal region in pocket 1; and His451 and Tyr621 located at pocket 2. The amino acids from packet 1 were also identified by both FTMap and PrankWeb. The amino acids Cys406, His525, Thr531, Pro548, Asn550, Trp554, and Asn580 were identified and shared by both PrankWeb and DeepSite, but not by the FTMap server (Table 2). The natural mutant variant LA0591which lacks RBLs, shows His205 identified by the three online servers (FTMap, PrankWeb and DeepSite) as involved in ligand binding (Figures 6B,D).

**TABLE 2:** Comparative assessment of hot-spot residues and ligand binding sites in LA3490 and bovine DNase.

LA3490C		
FTMap	PrankWeb/P2Rank	Deepsite
Arg615	Glu396, Thr397, Arg398, Val399	His533, Cys406
His533	Cys403, Pro405, Cys406, Met521	His525, Asn550
Cys403	Gln523, His525, Thr531, Gly532	Thr549, Gln523, Asn580
Gln486	His533, Val546, Pro548, Thr549	Thr531, Pro548, Trp554
Thr549	Asn550, Val551, Trp554, Phe562	His451, Tyr621
Gln523	Asn575, Glu577, Gly578, Ser579, Asn580	
Bovine DNase (3DN1)		
Asn170, His252, Tyr76, Arg111, Asn7, Asp251, His134, Glu39, Asp168	Glu39, Arg41, Asn7, Typ76, Glu78, Arg9, Ser110, Arg111, His134, Ser135, Ala136 Pro137, Asp168, Asn170, Cys173, Ser174 Try175, Thr203, Thr205, Thr207, Cys209 Thr211, Asp251, His252	His134, Try76, Glu78, Asp168, Pro137, Arg111, Asn7, Arg9
Characterized Active sites		
Bovine DNase (3DN1)	Arg9, Arg41, Tyr76, Glu78, His134, Asp168, Asp212 and His252	

Red color represent amino acids common resultant from FTMap, PrankWeb and Deepsite.

Blue color represent amino acids common resultant from PrankWeb and Deepsite.

Green represent amino acids common resultant from FTMap, and PrankWeb.

**FIGURE 7:**
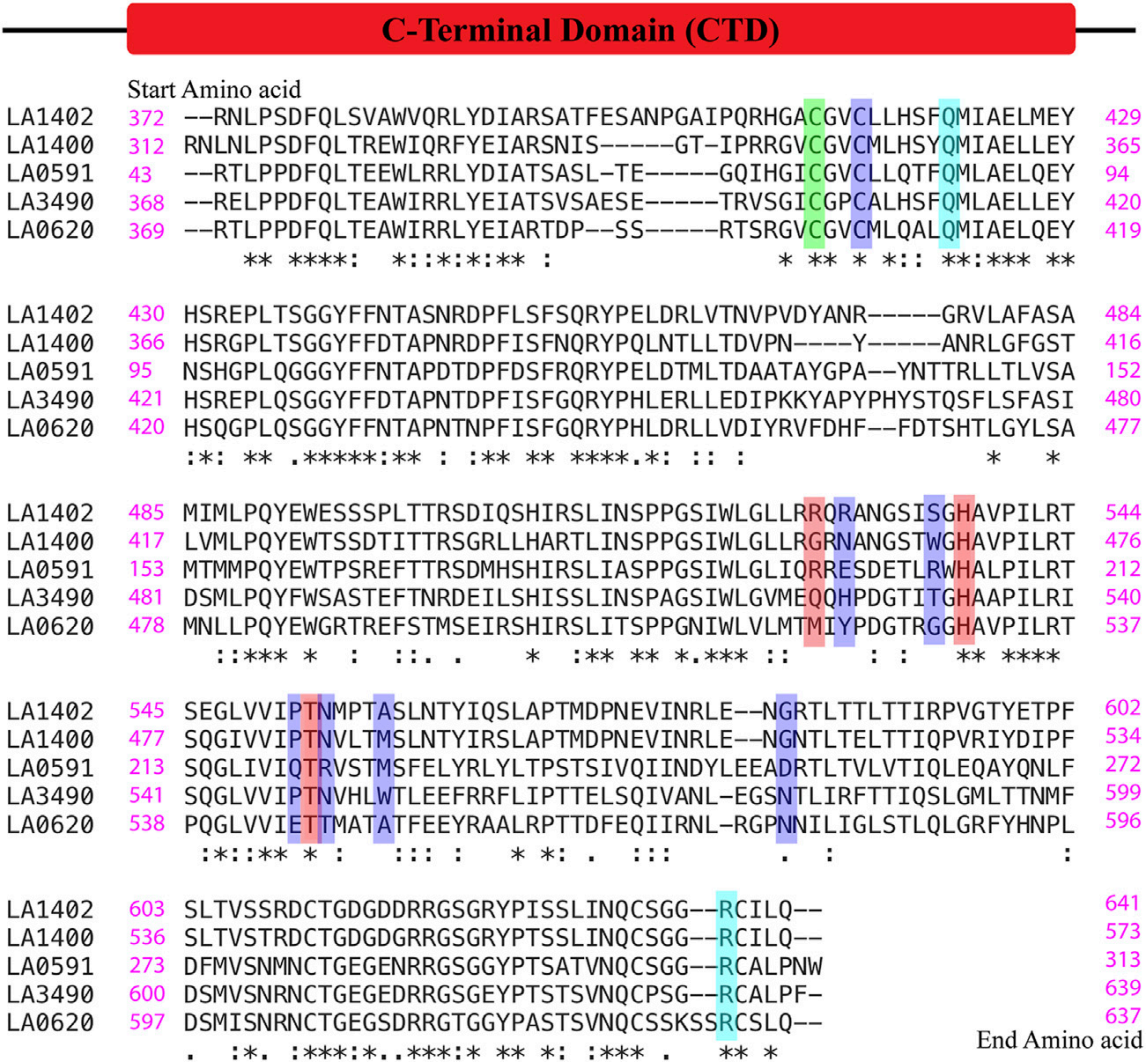
Multiple alignment sequence and conservation of hot-spot or ligand binding residues in VM proteins. The sequences of C-terminal domain of VM proteins (LA3490, LA0620, LA1400, LA1402, and LA0591, naturally lacks RBLs) were aligned using the Clustal Omega online server. (https://www.ebi.ac.uk/Tools/msa/clustalo/). The green color shows the conserved hot-spot residues (Cys403) resultant from FTMap and PrankWeb. The red color shows Gln523, His533 and Thr549 as common hot-spot residues resultant from the FTMap, PrankWeb and DeepSite algorithms. Only His533 and Thr549 was found to be conserved among the VM proteins. The ligand binding residues such as Cys406, His 525, Thr531, Pro548, Asn550, Trp554, Asn580 resultant from PrankWeb and DeepSite, shown in blue, were not conserved in the VM proteins except Cys406. FTMap-derived high binding residues Arg615, and Gln412 (cyan color) were conserved in the VM proteins and actively participate in docking with phosphate and magnesium ions.

This comparative study and multiple sequence alignment suggests that His533, Thr549, and Gln523 have high conservation, high confidence, and are actively involved in ligand binding; therefore, these amino acids could be useful for their functional study by site-directed mutagenesis (Figure 7).

Experimental validation from our pervious study shown that the full-length VM protein, unlike N-terminal domain, possess DNase activity. Phylogenetic and multiple sequence alignment suggested that CTD of LA3490 and bovine DNase (PDB: 3DNI) has closest similarity because of conserved active site residues (Chaurasia et al., 2022a). Therefore, the comparative study of identifying ligand binding sites in VM protein was validated with the 3D structure of bovine DNase (PDB: 3DNI) and its active site was superimposed with VM protein (Table 2). In bovine DNase, Asn7, Glu39, Tyr76, Arg111, Asp251, His134, Asp168, Asn170, and His252 were identified as hot-spot residues by FTMap and these amino acids were shared by PrankWeb and DeepSite (Table 2). The crystal structure of bovinike lectin (RBL1 and RBL2) domains and C-terminal toxin domains with DNase activity (Chaurasia et al., 2022a).

### C-terminal of VM protein-based DNase activity

**FIGURE 8:**
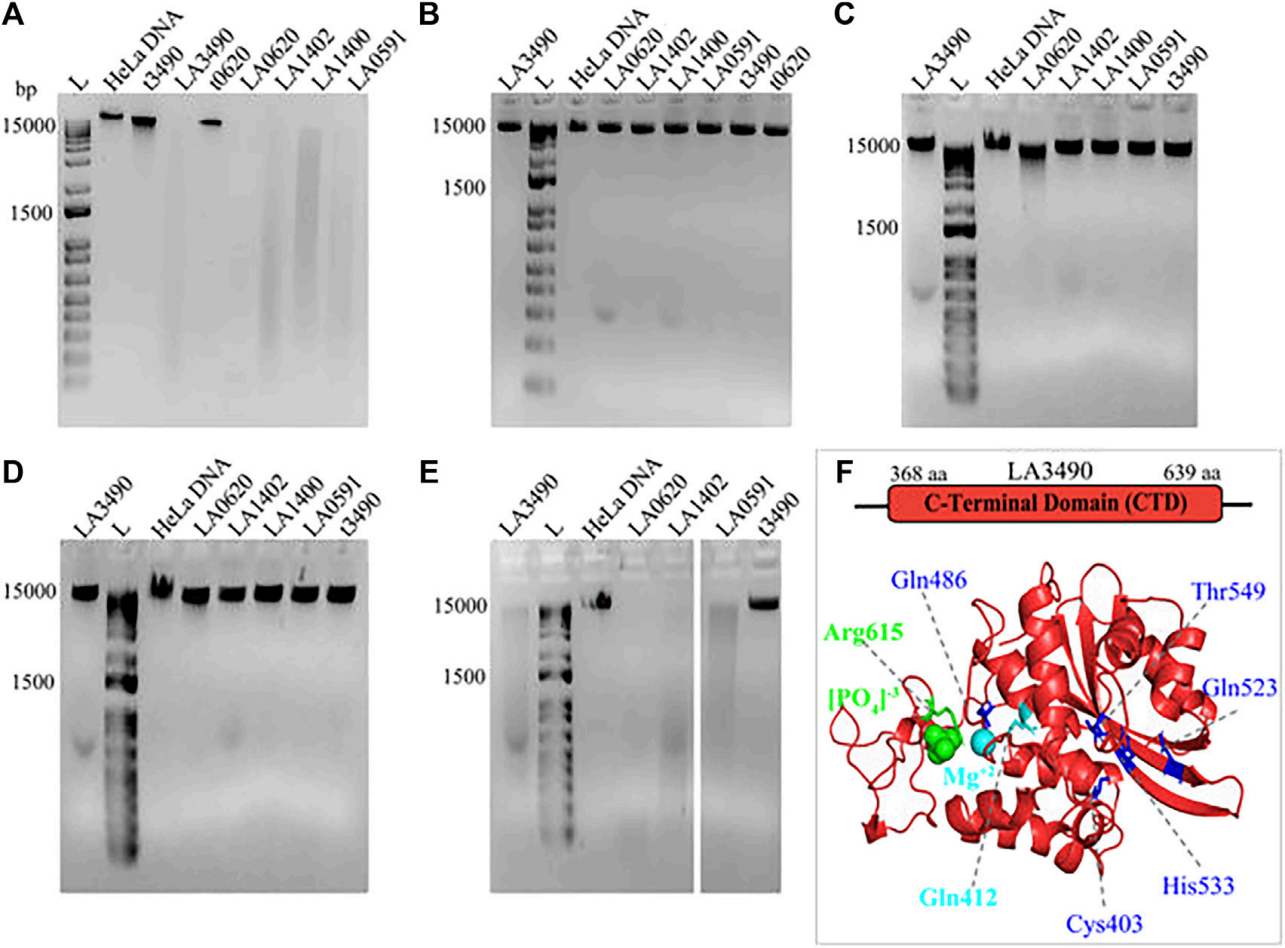
Demonstrating the effect of divalent cations on DNase activity of VM proteins. HeLa DNA (150 ng) were incubated with 30 nM of purified soluble recombinant VM proteins (t3490, LA3490, LA0620, LA1402, LA1400, and LA0591) in TM buffer (10 mM Tris pH-7.4) containing 3 mM MgCl_2_ **(A)**, absence of (divalent cation) MgCl_2_ **(B)**, presence of 2 mM ZnCl_2_ **(C)**, presence of 3 mM CaCl_2_ **(D)**, and presence of CaCl_2_ + 3 mM MgCl_2_ **(E)** for 30 min and samples were subjected to 1% agarose gel electrophoresis. The DNase activity by VM proteins is indicated by smearing and disappearance of DNA, whereas negative controls such as t3490, t0620 which does not encode for the functional CTD, had no such effect. **(F)** Docking study with CTD of LA3490 showing phosphate (green color spheres) and magnesium ion (cyan color sphere) interaction with Arg615 (binding energy −2.58 kCal/mol) and Gln412 (binding energy −0.95 kCal/mol) respectively. The residues having high binding energy shown in blue color.

The DNase activity of VM proteins is dependent on Mg^2+^; and the replacement of Mg^2+^ by Zn^2+^, Ca^2+^, or absence of Mg^2+^ abolishes the catalytic activity of VM proteins (Figure 8). The truncated 3490 or 0620 did not degrade the HeLa DNA because it encodes for ricin binding domain (RBL1) and lack the functional C-terminal domain therefore served as a negative control (Figure. 8A-E).

Mg^2+^interacts with hot-spot residue Gln412 and showed a binding energy of −0.95 kCal/mol; whereas phosphate interacts with hot-spot residue Arg615 with a predicted binding energy of -2.58 kCal/mol (Figure. 8F). Multiple sequence alignment shows Gln412 and Arg615 are highly conserved in VM proteins (Figure. 7), therefore approaches toward site-directed mutagenesis of Gln412 and Arg615 will help to reveal the essential catalytic residues involved in the hydrolysis of the substrate at C-terminal of VM proteins. The Predict protein algorithm, https://predictprotein.org online server, was used for further informative knowledge about structure-function and strengthened the computational analysis by showing the DNA binding domain within the C-terminal region of VM proteins (Supplementary Figure. S2).

## Discussion

Here we use the AI tool, AlphaFold, plus additional computational tools including molecular dynamic simulation, to delineate different functional domains, validate their stability and toxin active sites of leptospiral VM proteins. Recently published data supported the hypothesis that VM proteins are involved in the molecular and cellular pathogenesis of leptospirosis (Chaurasia et al., 2022a; Chaurasia et al., 2022b). This in silico analysis indicates further experimental directions to further explore mechanisms underlying severe leptospirosis at the molecular and cellular level.

The PF07598 gene family encodes multiple paralogs, which is expanded in serovars of L. interrogans, which have 12 or more paralogs of an ∼ 640 aa molecular weight VM protein belongs, and they encode single polypeptide transcribed from a single genetic locus, distinct from most other bacterial AB toxins. A RBL domain lacking variant (orthologs of LA0591) is uniquely found in L. interrogans, implying novel models of pathogenesis related to the lifestyle of the organism. Typical bacterial AB toxins have diverse mechanisms of action and may modify specific host targets by ADP-ribosylation, glycosylation, deamidation, de-adenylation, proteolysis, or acetylation. These modifications often result in an inactivation of the target, altered cell physiology, or may lead to necrotic or apoptotic cell death (73, 74). Leptospiral VM proteins uniquely have highly conserved, tandemly repeated, N-terminal trefoil-like lectin (RBL1 and RBL2) domains and C-terminal toxin domains with DNase activity (Chaurasia et al., 2022a). N-terminal structural similarities of VM protein with ricin B chain, conserved (QxW)_3_ motif and aromatic patches suggest that they belong to the lectin family. R-type lectins are members of a superfamily of proteins that have a carbohydrate-recognition domain (CRD) and binding functions for complex carbohydrates on glycoconjugates (glycoproteins, proteoglycans/glycosaminoglyca65ns, and glycolipids) such as ricin B chain (Cummings et al., 2017). The essentiality of full-length canonical VM proteins binding to a cell-surface host receptor is key to understanding one aspect of host-pathogen interactions. The well-studied ricin B chain (RTB) is a galactose-specific lectin and contains two identical sugar binding sites, preferably to oligosaccharides (Frankel et al., 1996). One terminal galactose is bound by binding site 1 (W37) of RTB while the other terminal galactose can bind to the binding site 2 (Y248) of another molecule of RTB without steric hindrance, to make a strong hydrophobic interaction which stabilizes the protein–sugar complexes (Sphyris et al., 1995). Therefore, with reference to the ricin B chain, we hypothesize that the deletion of aromatic patches or replacement of tyrosine and tryptophan in the (QxW)_3_ or _158_YGY_160_ motifs of VM protein would likely destabilize the basic structure of the *ß*-trefoil fold. This would lead to a testable hypothesis that the discovery of novel small molecules would inhibit the binding of VM proteins to the host cell surface and may block VM protein-mediated damage to target cells.

*Leptospira*-secreted cytotoxins, perhaps in addition to other *Leptospira-*associated cellular components or other undefined toxins, may cause tissue damage leading to the pathogenesis of leptospirosis, at the level of endothelial or epithelial cells. This mechanism may contribute to renal ischemia, tubular necrosis, disseminated intravascular hemolysis, and severe pulmonary hemorrhages, eventually leading to dysfunction of multiple organ failure (Adler and de la Pena Moctezuma, 2010; Haake and Levett, 2015). Antibody/complement-containing immune complexes have been observed to be deposited on the lung alveolar basement membrane in severe leptospirosis in the guinea pig model of infection as well as in humans; it remains unproven whether such immune complex deposition is a primary or secondary effect of severe disease pathogenesis (Lai et al., 1982; Nally et al., 2004; De Brito et al., 2013a; De Brito et al., 2013b). The current study used complementary computational approaches to identify functional motifs, binding, translocation, and catalytic active site residues. Our analyses suggest that VM proteins are multi-functional by virtue of globular domains similar to plant N-terminal ricin B chain like lectins (binding domain), bacterial CARDS toxin (translocation domain), and C-terminal mammalian DNase domain (enzymatic domain). Future studies may determine the cellular target, binding efficiency, and substrate specificity and therapeutic anti-toxin agents plausibly will ameliorate the dire clinical manifestations of severe leptospirosis.

In the process of evolution for survival, pathogenic bacteria have developed an array of sophisticated virulence factors/toxins (Kumar et al., 2019) involving host-pathogen interactions; and the secretion of toxins that damage host tissue (as well as potential environmental reservoirs such as free-living amoebas) is well established as vital to the infectious processes of many bacterial pathogens (Collier, 1975; Cook et al., 2001; Spencer, 2003; Nelson et al., 2009; Nigam and Nigam, 2010; Hamond et al., 2019). Despite our considerable knowledge of the clinical manifestations, histopathological responses, and pathogenesis of leptospirosis, there are inadequate studies and reports describing the role of leptospiral toxins in the pathogenesis. Identification of the PF07598 gene family in pathogenic *Leptospira* opened an area to study the secretory exotoxins and their mode of action in the pathogenesis of leptospirosis. Our recent study suggests that these VM proteins encode novel R-type lectin domain-containing cytotoxins. The N-terminal domain of the VM proteins is structurally similar to the ricin B chain. Recombinant VM proteins exerted rapid and potent cytopathic effects on cultured HeLa cells. Recombinant VM-mCherry tagged proteins bind to the HeLa cell surface, translocate to the nucleus, and rapidly lead to chromosomal fragmentation and cell death (Chaurasia et al., 2022a). Phylogenomic and AlphaFold-predicted structural analyses of the PF07598 gene family established the robust statistical support that VM proteins contain conserved motifs mediating attachment, internalization, nuclear localization, and chromosomal fragmentation occurring *via* C-terminal nuclease activity (Chaurasia et al., 2022a). An *in vivo* vaccination study in mice revealed that VM proteins prevented death and led to a 10^4^–10^5^-fold lower liver/kidney bacterial load in a susceptible mouse model (Chaurasia et al., 2022b). These data collectively show that leptospiral VM proteins bind to cells and possess cyto/genotoxic activity, are candidates for playing pivotal roles in leptospirosis pathogenesis and could be used as therapeutic agents. The current study using computational approaches supports the hypothesis that VM proteins are toxins and play a pivotal role in leptospirosis disease.

Furthermore, the superimposition of RBL2 of VM proteins and CARDS (D3 domain) with RMSD -1.218 Å and six conserved tryptophan in their aromatic patch, thus strengthening the RBL2 function as a translocation domain in VM proteins for internalization into the host cell. Mutagenesis of residues 571–591 aa of CARDS toxins, which is integral to the proper folding of D3 and formation of its aromatic patch, inhibited internalization by HeLa cells; and therefore, suggests that CARDS toxin entry into host cells is mediated by the D3 domain (Becker et al., 2015; Ramasamy et al., 2018). We hypothesize that the mutation in these conserved tryptophan/aromatic patches would abolish the internalization of the toxin into host cells. The fundamental study of these motifs or carbohydrate binding domain could be achieved by mutagenesis, and glycan microarrays and further validation by surface plasma resonance (SPR), to examine glycan-protein (host-pathogen) interactions or in the identification of host receptors and innate immune receptors (Geissner et al., 2019).

The disulfide bond-dependent secondary structure of bacterial AB toxins is essential for their functional roles (Hogg, 2003). Reduction of disulfide bonds in the A chain and B chain of ricin decreases its toxicity in mice and its ability to inhibit protein synthesis of HeLa cells (Lappi et al., 1978). Ricin A chain (RTA) contains two cysteine residues (Cys171 and Cys259), and Cys 259 forms the interchain disulfide bond of ricin holotoxin with Cys4 of ricin B chain (RTB). Disruption of this disulfide bond by site-directed mutagenesis (cysteine at 259 aa in ricin replaced with alanine) within the A chain reduces the cytotoxicity (Mohanraj and Ramakrishnan, 1995), and the introduction of a new disulfide bond into ricin A chain decreases its cytotoxicity (Argent et al., 1994). Based on the structural superimposition of ricin with VM proteins, the disulfide bond at position 353 aa—608 aa (4 aa—259 aa in ricin) is believed to be crucial for C-terminal proteolysis or hydrolysis in endosomes, where the biologically active C-terminal domain is subsequently released into the cytosol and is translocated to the nucleus. However, CARDS toxins encode for six cysteines residues at amino acid positions 230, 247, 324, 406, 425, and 548; and the disulfide bond formation between residues C230 and C247 is essential for its cytotoxicity. Mutagenesis study revealed that the disulfide bond protects the ADPRT (D1) domain of CARDS toxin from proteases; and a disrupted disulfide bond does not affect cell binding, internalization, and intracellular trafficking (Balasubramanian et al., 2019). For the A chain of shiga toxin, a disulfide bond stabilizes the toxin subunit after protease cleavage in the endosome or trans-golgi network (Garred et al., 1995; Tam and Lingwood, 2007); however, a disulfide mutant of Shiga toxin was more susceptible to proteolytic degradation and less cytotoxic to cells (Garred et al., 1997). Likewise, in pertussis toxin, the reduction of the disulfide bond alters its conformation, which is required for the toxin to exhibit NAD glycohydrolase and ADPRT activities (Moss et al., 1983; Burns and Manclark, 1989). In diphtheria toxin and cholera toxin, the reduction of the disulfide bond results in the release of an active fragment from the endosomes into the cytosol (Falnes et al., 1994; Collier, 2001; Tsai et al., 2001; Sandvig and van Deurs, 2002). The significance of disulfide bonds in bacterial toxins and their role in pathogenesis strengthen the importance of computational prediction of RBLs and disulfide bond architecture of the PF07598 gene family (Figure 4). The approach towards site-directed mutagenesis of cysteine residues or engineering new disulfide bonds in VM proteins could be used to reduce the cytotoxicity, and hence could be used as a vaccine candidate. Taken together, our current study hypothesizes the critical role of the disulfide bond in the activation of VM toxin and subsequent cytopathological events.

In bovines and humans, His134 and His252 and their hydrogen-bond pairs Glu 78 and Asp 212 are crucial for functional DNase I activity (Pan et al., 1998); and mutations at any of the four catalytic amino acids (His 134, His 252, Glu 78, and Asp 212) drastically reduced the hydrolytic activity of DNase I (Pan et al., 1998). The list of shared amino acids in VM proteins by three independent servers FTMap, PrankWeb, and DeepSite, and the identification of ligand binding sites of bovine and human DNase which overlap with an active site, suggest that these machines learning based algorithms are reliable to screen the hot-spot residues/ligand binding sites/active sites in VM proteins.

The PF07598 gene family encodes multiple VM proteins and transposon mutagenesis suggests that LA0589 contributes to lethal disease in hamsters (Murray et al., 2009; Truong and Coburn, 2011). Rapid and specific gene silencing/targeted knockout of PF07598 genes in a combination of CRISPR/Cas9 and Non-homologous End-Joining (NHEJ) System, could aid studies in the functional role of individual VM proteins (Fernandes and Nascimento, 2022), which would explain their expanded repertoire in *L. interrogans*, *L. kirschneri* and *L. noguchii* compared to other pathogenic *Leptospira* species (Lehmann et al., 2013; Fouts et al., 2016).

**FIGURE 9:**
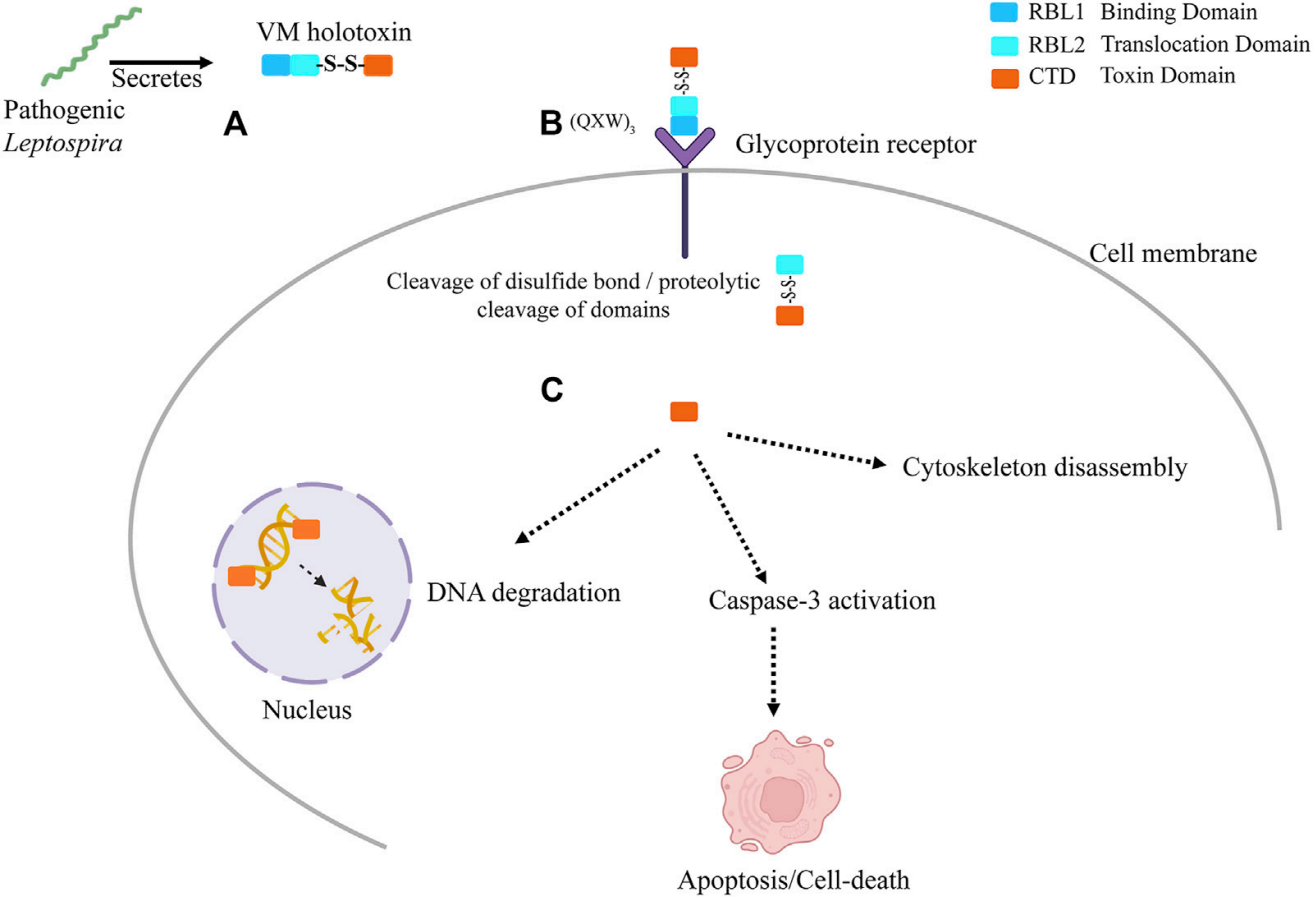
The graphical presentation of proposed hypothetical model: VM proteins have myriad functions that contribute to leptospirosis pathogenesis. **(A)** Pathogenic *Leptospira* secrete exotoxins. These exotoxins are multi-globular domain AB-toxins encoded by a single gene. The N-terminus possesses ricin B-like lectin domains (RBL1 and RBL2) with multiple disulfide-bonds and functional DNase domains at the C-terminus containing a single disulfide bond. **(B)** The VM protein binds to terminal galactosyl-containing glycoproteins of the host cell surface *via* RBL1 domain comprising carbohydrate binding characteristic lectin family sequence motif (QxW)_3_ and aromatic patches, located at N-terminal and then translocated inside the host cell plausibly by RBL2 translocation domain. **(C)** The VM protein must undergoes proteolytic cleavage, which is essential to produce the functional domains. The cleavage of disulfide bond further releases the functional toxin domain in the cytosol. Nuclear localization signal direct the toxin domain into the nucleus where it disrupts the chromosomal integrity. The secondary effects such as cytoskeleton disassembly and caspase-3 activation plausibly led to apoptosis and cell-death.

Our previous published validated *in vitro* data (Chaurasia et al., 2022a) and current *in silico* study suggest the potential role of VM proteins pathogenesis of leptospirosis. Here, we propose a hypothetical model (Figure 9) showing the plausible mechanistic pathway of VM proteins and their role in disease severity (A) LA3490 secretory protein enters the cell *via* RBL1 domain possess (QxW)_3_ motif which binds to the glycoprotein receptor present on cell membrane (B) The putative enzymatic domain internalizes in the cytosol plausibly by RBL2 translocation domain; C) Because multi-globular VM proteins are encoded by a single gene locus, unlike other AB toxins therefore the proteolytic cleavage is a vital step to produce the functional domains. Further cleavage of disulfide bind (353 aa- 608 aa) between RBL2 and C-terminal toxin domain is essential to release the functional domain in the cytosol. Nuclear localization signal translocates the enzymatic domain into the nucleus, leading to nuclear fragmentation, caspase-3 activation, apoptosis, and cytoskeleton disassembly. The comprehensive experimental approach towards the proposed hypotheses would likely to provide insights of how the VM protein family exert the pathogenetic mechanisms during mammalian infection and manifesting leptospirosis severity.

## Conclusion

Here we use computational modelling to gain insights into understanding leptospiral exotoxins and to generate testable hypotheses for future experimentation to define the molecular pathogenesis of leptospirosis. The PF07598 gene family encodes multi-globular domains with distinct functions. Each of the domains has specific structures and sequence similarities indicating accretion from diverse origins, such as the N-terminal domain similar to plant-derived ricin B chain, a translocation domain similar to the D3 domain of bacterial CARDS toxin, and a C-terminus as a functional catalytic DNase domain. We discussed the significance of domains of the VM protein and provided the informative approach for site-directed mutagenesis of key motifs and residues, a step closer to their characterization. VM proteins have the potential as targets small molecule drugs, mAb biologics, and vaccine interventions to prevent and treat severe leptospirosis. The current insights have opened a window to study the dynamic view of the mechanism prevailing leptospiral pathogenesis.

## Data Availability

The original contributions presented in the study are included in the article/Supplementary Materials, further inquiries can be directed to the corresponding authors.
