# Supplementary figures and images for: In silico prediction of molecular mechanisms of toxicity mediated by the leptospiral PF07598 gene family-encoded virulence-modifying proteins
Source: Front Mol Biosci. 2023 Jan 23;9:1092197. doi: 10.3389/fmolb.2022.1092197 (PMC9900628; doi:10.3389/fmolb.2022.1092197)

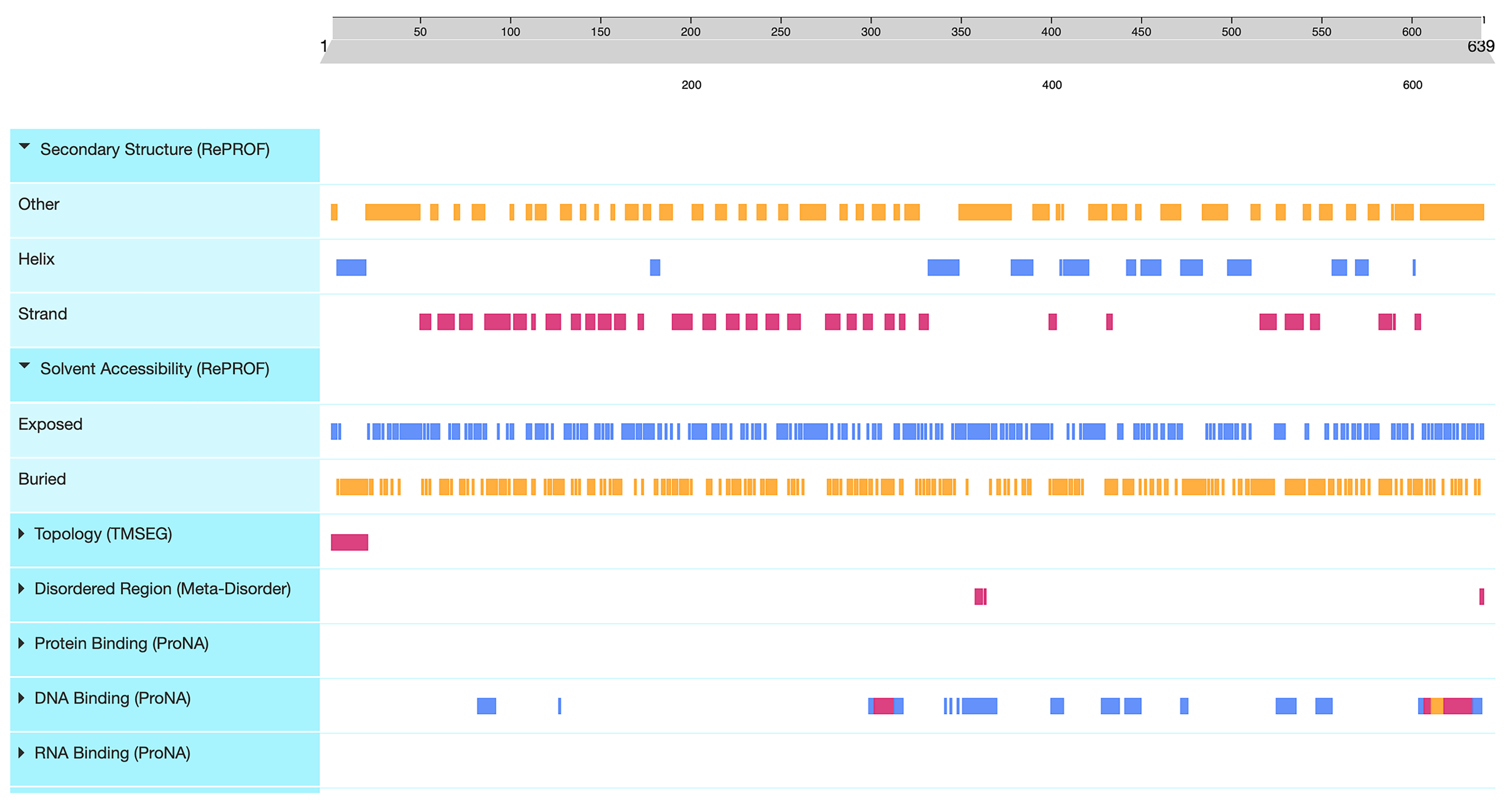

Supplement: Supplementary file 3 [file Image2.TIFF]
